# Effect of Rho-kinase inhibition on complexity of breathing pattern in a guinea pig model of asthma

**DOI:** 10.1371/journal.pone.0187249

**Published:** 2017-10-31

**Authors:** Saeed Pazhoohan, Mohammad Reza Raoufy, Mohammad Javan, Sohrab Hajizadeh

**Affiliations:** Department of Physiology, Faculty of Medical Sciences, Tarbiat Modares University, Tehran, Iran; Forschungszentrum Borstel Leibniz-Zentrum fur Medizin und Biowissenschaften, GERMANY

## Abstract

Asthma represents an episodic and fluctuating behavior characterized with decreased complexity of respiratory dynamics. Several evidence indicate that asthma severity or control is associated with alteration in variability of lung function. The pathophysiological basis of alteration in complexity of breathing pattern in asthma has remained poorly understood. Regarding the point that Rho-kinase is involved in pathophysiology of asthma, in present study we investigated the effect of Rho-kinase inhibition on complexity of respiratory dynamics in a guinea pig model of asthma. Male Dunkin Hartley guinea pigs were exposed to 12 series of inhalations with ovalbumin or saline. Animals were treated by the Rho-kinase inhibitor Y-27632 (1mM aerosols) prior to each allergen challenge. We recorded respiration of conscious animals using whole-body plethysmography. Exposure to ovalbumin induced lung inflammation, airway hyperresponsiveness and remodeling including goblet cell hyperplasia, increase in the thickness of airways smooth muscles and subepithelial collagen deposition. Complexity analysis of respiratory dynamics revealed a dramatic decrease in irregularity of respiratory rhythm representing less complexity in asthmatic guinea pigs. Inhibition of Rho-kinase reduced the airway remodeling and hyperreponsiveness, but had no significant effect on lung inflammation and complexity of respiratory dynamics in asthmatic animals. It seems that airway hyperresponsiveness and remodeling do not significantly affect the complexity of respiratory dynamics. Our results suggest that inflammation might be the probable cause of shift in the respiratory dynamics away from the normal fluctuation in asthma.

## Introduction

Normal breathing reveals variable dynamics, both in respiratory rate and volume [1] that arises from the complex interaction between several internal [2] and external environmental changes [3, 4]. Respiratory system is a highly dynamic physiological network that continuously fluctuates under non-equilibrium steady-state conditions [5] in order to adapt to different variety of stimuli [6, 7]. Alteration of these dynamic properties is associated with diseased states [5]. Asthma is a complex disease characterized by a fluctuation in clinical symptoms and behavioral manifestations over time [8]. Such variable behavior is associated with greater distance from the steady-state equilibrium that shifts respiratory dynamics toward an attenuated level of complexity [7, 9].

Dames and colleagues reported that reduced complexity of air flow pattern is related to the severity of airway obstruction [10]. Also, increased variability and decreased correlations of peak expiratory flow fluctuations have been shown to be related to poor asthma control [11]. In addition, increased temporal self-similarity and variability of peak expiratory flow (PEF) fluctuations is associated with failure of treatment [12]. Our previous studies have demonstrated decreased long-range correlations, increased regularity and reduced sensitivity to initial conditions of both inter-breath interval (IBI) and respiratory volume (RV) fluctuations in patients with asthma, particularly in uncontrolled state [9]. Moreover, we found increased memory length of respiratory pattern in uncontrolled asthma [13]. Therefore, complexity analysis of respiratory signals can provide a new approach for prediction of asthma episodes, controllability and severity [11, 14, 15].

The pathophysiological basis of alteration in breathing pattern complexity in asthma has remained unknown. However, understanding the underlying mechanisms of such variable behavior may provide new insights to develop novel interventions and therapeutic strategies for management of asthma [9, 16].

Major pathophysiological features of asthma include airway hyperresponsiveness (AHR), remodeling, inflammation, and mucus hypersecretion [17–19]. Several lines of evidence suggest that Rho-kinase is involved in pathophysiology of asthma [20–23]. Previous studies have reported that repeated challenge of allergen induces the expression of Rho-kinase at both mRNA and protein levels within the airway smooth muscle cells [24, 25]. It also increases the translocation of RhoA protein to cell membrane in these cells [26]. Kasahara et al., have shown that challenging mice that are haploinsufficient for Rho-kinase with OVA can abrogate development of AHR [27]. Inhibition of Rho-kinase in animals challenged with allergen has also been shown to decrease AHR, airway inflammation and remodeling including reduced volume fractions of collagen, elastic fibers and smooth muscle actin [25, 28, 29]. Since Rho-kinase signaling pathway plays a cardinal role in pathophysiology of asthma, it is likely that Rho-kinase signaling contributes to the complexity of respiratory dynamics in asthmatic patients. Taken together, in the present study, we investigated the effect of Rho-kinase inhibition on complexity of respiratory dynamics in a guinea pig model of asthma.

## Methods

### Animals

Pathogen-free male Dunkin Hartley guinea pigs (Razi Institute, Karaj, Iran) weighing between 350–500 g, were housed in plexiglas breeding cages with free access to food and water. Animals were kept in a colony room with constant temperature and under a standard 12:12 h light: dark cycle. All protocols were approved by the "Ethics Committee of Faculty of Medical Sciences, Tarbiat Modares University" based on the ‘‘NIH Guide for The Care and Use of Laboratory Animals”. All experiments were performed at the same time of the day (10am-2pm) to minimize the impact of circadian rhythm.

### Sensitization protocol

The chronic model of asthma was induced by a modified sensitization protocol [29]. For this purpose, animals were placed in a plexiglas box (40 × 20 × 20 cm) and exposed to the aerosol generated by a compressor nebulizer (Pari, Starnberg, Germany) for 15 minute every 3 days for a period of 5 weeks (totally 12 times). Ovalbumin (OVA) was applied with incremental doses (1 to 10mg/ml, diluted in NaCl 0.9%) to prevent the development of drug tolerance [30].

### Animal groups and experimental protocols

Animals were randomly divided into three groups (n = 10 for each group): *OVA group*, animals received OVA aerosol using sensitization protocol; *OVA+Y-27632 group*, animals were subjected to inhalation of Rho-kinase inhibitor (1mM) (Y-27632, Apexbio, USA) 10 min before the eight last OVA exposures for 6 min; *control group*, animals only received saline aerosol as the same protocol used for OVA administration. In addition, there was an *OVA+saline group* in which animals received saline before OVA administrations similar to the protocol used for the application of Rho-kinase inhibitor in *OVA+Y-27632 group*.

Since changes in airway conductance and eosinophil infiltration reaches to the maximum in 72h following antigen exposure in Guiana pigs, we examined AHR and inflammation 72h after the last antigen challenge [31–33].

### Recording of respiration

**Fig 1:**
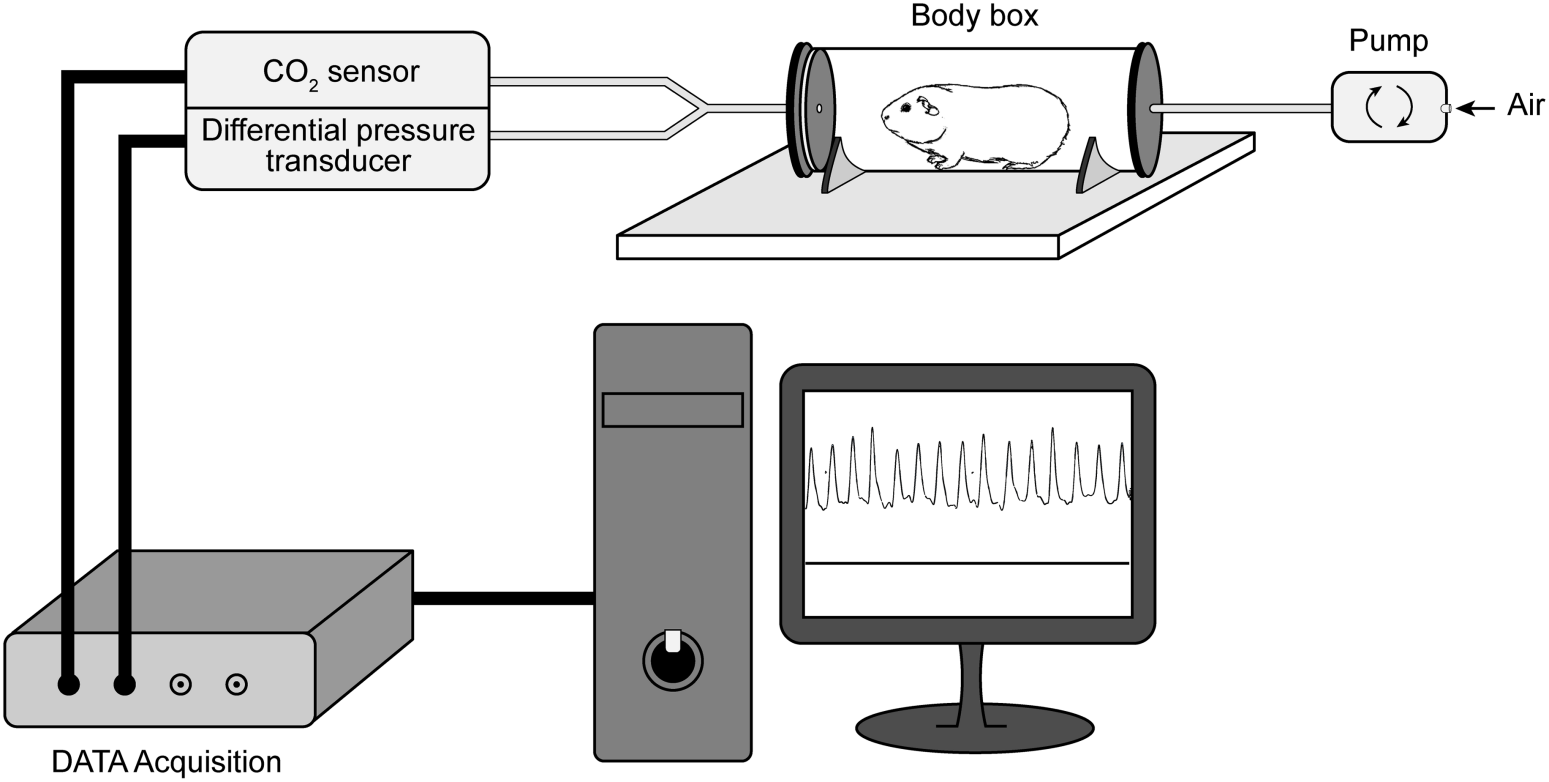
Diagram of the custom-built whole-body plethysmograph. The apparatus included a cylindrical chamber made of transparent Plexiglas. Room air was continuously pumped (4 L/min) into the chamber. A plastic Y-connector was connected to chamber's outlet. One of the exit ports was linked to one input of a differential pressure transducer, and the other input being exposed to the room air. Another port Y-connector was connected to a one input of CO2 sensor. The other input of the CO2 sensor was opened to the room air. The respiratory signals were acquired at the sampling rate of 1 kHz.

The respiration of conscious guinea pigs was recorded one day before aerosol challenge and 48h after the last aerosol challenge using whole-body plethysmography. Briefly, a custom-built whole-body plethysmograph was used in this study. The apparatus included a cylindrical chamber made of transparent plexiglas (i.d. 90 mm, length 300 mm, volume 2 L, and wall thickness 5 mm) with oppositely aligned inlet and outlet ports in each wall. Two 2 mm male luer lock connectors allowed input and output polyethylene tubing lines to be tightly connected to the chamber. Room air was continuously pumped into the chamber at a controlled flow rate (4 L/min) in order to prevent any rise of CO2 in the plethysmograph. A plastic Y-connector was placed at a distance of 10 cm from the chamber's outlet, and one of the exit ports was linked to one input of a differential pressure transducer (MPXV7002DP, Freescale Semiconductor Inc.), the other input being exposed to the room air. The plethysmograph chamber was also connected to one input of a CO2 sensor (MG811, Sandbox Electronics Inc.) by means of polyethylene tubing linked to second exit port of the Y-connector. The other input of the CO2 sensor was opened to the room air (Fig 1).

In order to allow the animals to get acclimated to recording chamber, another transparent plexiglas cylinder of the same size as the plethysmograph chamber was placed in all home cages. In addition, animals were placed to recording box one hour per day for 7 days prior to the beginning of experiments. On the test day, animals were gently guided into the chamber without being forced. Each experiment consisted of a 10-min initial recording period (as the acclimatization time) which was then followed by a 60-min main recording period.

### Analysis of breathing pattern

**Fig 2:**
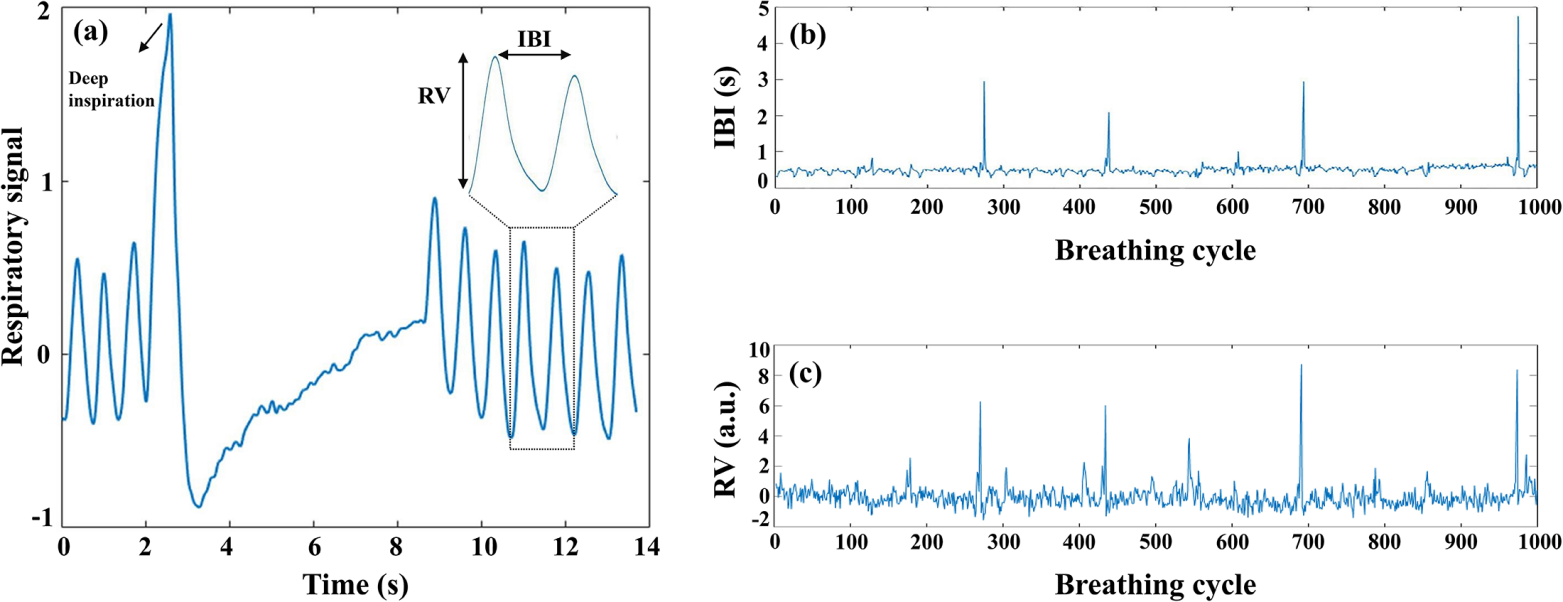
Respiratory pattern in a representative guinea pig. (a), Representative fragments of the respiration signal display the inter-breath interval (IBI) and respiratory volume (RV), as well as deep inspiration. Original IBI (b) and RV (c) time series during 20 min of respiration in a representative guinea pig.

The respiratory signals were acquired at the sampling rate of 1 kHz using a Powerlab system (AD Instruments, Australia) and displayed on ChartPro 7.0 software. We selected 20 min of respiratory signal with the lowest artifact for breathing pattern analysis. Time-series of interbreath interval (IBI) and respiratory volume (RV) were computed using a custom program written in MATLAB software. The peak of each breath signal was identified and peak-to-peak time intervals and the amplitude of peaks were thought-out as the IBI and RV series, respectively (Fig 2).

#### Sample entropy (SampEn)

Irregularity of respiratory pattern was quantified by calculating sample entropy as an index representing the degree of complexity. SampEn is the negative natural logarithm of the conditional probability that two sequences similar for *m* points remain similar at the next point within a tolerance (*r*), without allowing self-matches [34, 35]. Different values of parameters (*m*, *r*, *N*) are used for entropy analysis of physiological time-series, where *N* is the length of the time-series, *r* is the tolerance for accepting matches, and *m* (embedding dimension) is the length of sequences to be compared [34]. In our analysis, we computed SampEn of IBI and RV by the values of 2 for *m* and 0.2 for *r* [9], using MATLAB code available from the physionet (http://www.physionet.org).

#### IBI and RV cross-sample entropy (Cross-SampEn)

Cross-sample entropy is a method for analyzing two related time-series to measure the degree of their asynchrony [34, 35]. A higher degree of asynchrony demonstrates fewer sub-pattern matches, as quantified by larger Cross-SampEn values. On the contrary, lower values are demonstrative of stronger synchronization [34, 35]. We calculated Cross-SampEn after setting the values of *m* (2) and *r* (0.2) [9] for quantifying asynchrony between IBI and RV time-series, using customized MATLAB code available from the PhysioNet. (http://www.physionet.org).

#### Detrended fluctuation analysis (DFA)

The fractal-like temporal structure of a time-series can be evaluated by DFA method [36]. In this technique, the root mean square of the oscillation of the integrated and detrended data is measured within time windows of various sizes and then plotted against window size on a log–log scale. A linear relationship between log (fluctuation) and log (window size) demonstrates the presence of self-similar time-series [36]. The slope of this line is the α scaling exponent. This parameter indicates the correlation within the time-series regardless of the scale.

### Measurement of airway responsiveness

Airway responsiveness to methacholine (MCh) was investigated 72 h after the last aerosol exposure. For this purpose, animals were anaesthetized with intraperitoneal injection of urethane (1.5 g/kg) and then they were subjected to tracheostomy and mechanical ventilation with an Inspire Advanced Safety Volume Controlled Ventilator (Harvard Apparatus, Holliston, MA) at a tidal volume of 1.0 ml/100g body weight and frequency of 60 breaths/min. Afterwards, animals were paralyzed by intraperitoneal injection of pancuronium bromide (0.2 mg/kg).

After five min of ventilation and stabilization, animals were inhaled with physiological saline for one minute to measure the basal airway pressure. For evaluation of airway responsiveness to MCh (Provocholine, Canada), incremental doubling doses of MCh from 0.2mg/ml to 1.6 mg/ml were administered via compressor nebulizer (Pari, Starnberg, Germany), for one min at 5-min intervals. The airway resistance is defined as the percentage of airway pressure evokes by MCh to basal pressure (100%) obtained by saline administration. Afterwards, animals were sacrificed by exsanguination.

### Bronchoalveolar lavage fluid (BALF) analysis

Three ml of sterile physiological saline was slowly instilled and gently withdrawn into the syringe (3 times) through the endotracheal tube with retrieval rates of about 60%. Equal volume of all samples (1.5 mL) was centrifuged at 1,200 rpm for 5 min at 4°C. Supernatant was discarded and cells were washed thrice in RPMI Medium. Total cells and eosinophils counts were performed on BAL cytospin samples prepared by cytocentrifugation (cytospin 3, Shandon Instruments, Pittsburgh, PA) and stained with Giemsa stain [37].

### Histological assessment

Animals’ lung were immediately removed after BALF collection and left lung was fixed in formalin 10% [38–41]. Then, 5-micrometer sections were obtained using a microtome (Leica RM 2135). The lung sections were stained with haematoxylin and eosin (H&E) to identify the airway infiltration of inflammatory cells, periodic acid-Schiff (PAS) to detect mucus producing epithelial cells, and Masson's trichrome to evaluate the distribution of collagen within the lungs. Histological alterations were assessed around bronchioles and larger airways using light microscopy. The right lung was fixed with 4% paraformaldehyde and prepared for assessment of smooth muscle actin (α-SMA). The lung cryosections were incubated overnight at 4°C with the primary antibody against α-SMA (Abcam, at 1∶100 dilution). Following immunofluorescence staining protocol, sections were incubated with the appropriate secondary antibody (goat anti-rabbit IgG-FITC, 1:200, Santa Cruz). Sections were counterstained with DAPI (blue) and imaged using fluorescence microscopy (Olympus BX51 TRF, USA). In the qualitative histological analysis, all sections were evaluated by two blinded observers. The intra- and inter-observer errors were 9% and 11%, respectively.

### Quantitative real-time RT-PCR

We used real-time RT-PCR to investigate the mRNA expression of IL-13, as a Th-2 cytokine which is involved in allergic diseases and TNF-α, as a pro-inflammatory mediator, in experimental groups [42, 43]. GAPDH RNA was used as an internal control. Approximately 100 mg of lower lobe of the right lung was harvested, immediately frozen in liquid nitrogen and then stored in −80 degree Celsius freezers. Frozen lungs were crushed in liquid nitrogen with a mortar and pestle. Total RNA was extracted using TRIzol reagent. Single-stranded cDNA was synthesized using cDNA reverse transcription kit (Aryatous Biotech, Tehran, Iran). Oligonucleotide primers used for PCR were as follows:

1. Guiana pig IL-13, XM_003464536.1, PCR product size: 96bp. Forward: GGACCCAGAAGATACTGAG, Reverse: ACTCAGCCACCTCGATCTTG.
2. Guiana pig TNF-α, NM_001173025.1, PCR product size: 105bp. Forward: TGAGGTCAACCTGCCTCAGTA, Reverse: AGAAGGGATGGATGGAGAGAC.
3. Guiana pig GAPDH, NM_001172951.1, PCR product size: 189bp. Forward: CCAGGGCTGCTTTCATGTCT, Reverse: GATCTCGCTCCTGGAAGATGG.

PCR reactions comprised 3 μL of cDNA, 1 μL of Forward and Reverse oligonucleotide primers (10 pmol/ml), and 10 ml of SYBR Green master-mix reagent (Ampliqon, Denmark) in a final volume of 20 ml. Real-time PCR was carried out via the following protocol: 10 min incubation at 95°C, followed by 45 cycles of denaturation step at 95°C for 45 sec, annealing step at 60°C for 45 sec, extension step at 72°C for 45s and followed by a final extension step for 10 minute at 72°C.

### Statistical analysis

The GraphPad Prism V6.07 (GraphPad Software, San Diego, CA) was used for statistical analysis of data. The differences in airway pressure for groups were compared by repeated measure two-way ANOVA followed by Bonferroni's post hoc test. For breathing pattern, BALF cells, and mRNA expression comparison, the significance of differences among groups were calculated using a one-way ANOVA with a Bonferroni post hoc test or a Kruskal–Wallis non-parametric test with a Dunn’s post-test. Data are presented as mean ± standard error of the mean (SEM). P-values less than 0.05 were considered statistically significant.

## Results

### Effect of Rho-kinase inhibition on airway remodeling and responsiveness

**Fig 3:**
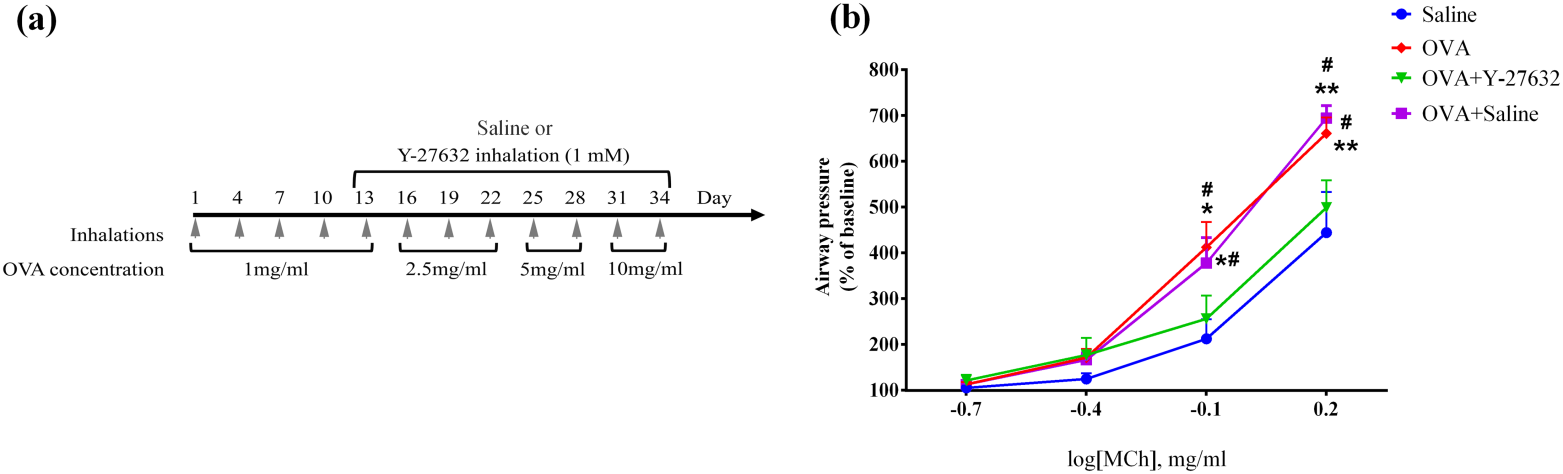
Effect of Rho-kinase inhibition on hyperresponsiveness. (a), Experimental protocols; (b), airway hyperresponsiveness in response to methacholine. Data are presented as percent of baseline and expressed as mean ± SEM (n = 6). * p<0.05 and ** p<0.01 compared to saline group; # p<0.05 compared to OVA+Y-27632 group; analyzed by repeated measure two-way ANOVA, with the Bonferroni post-test.

**Fig 4:**
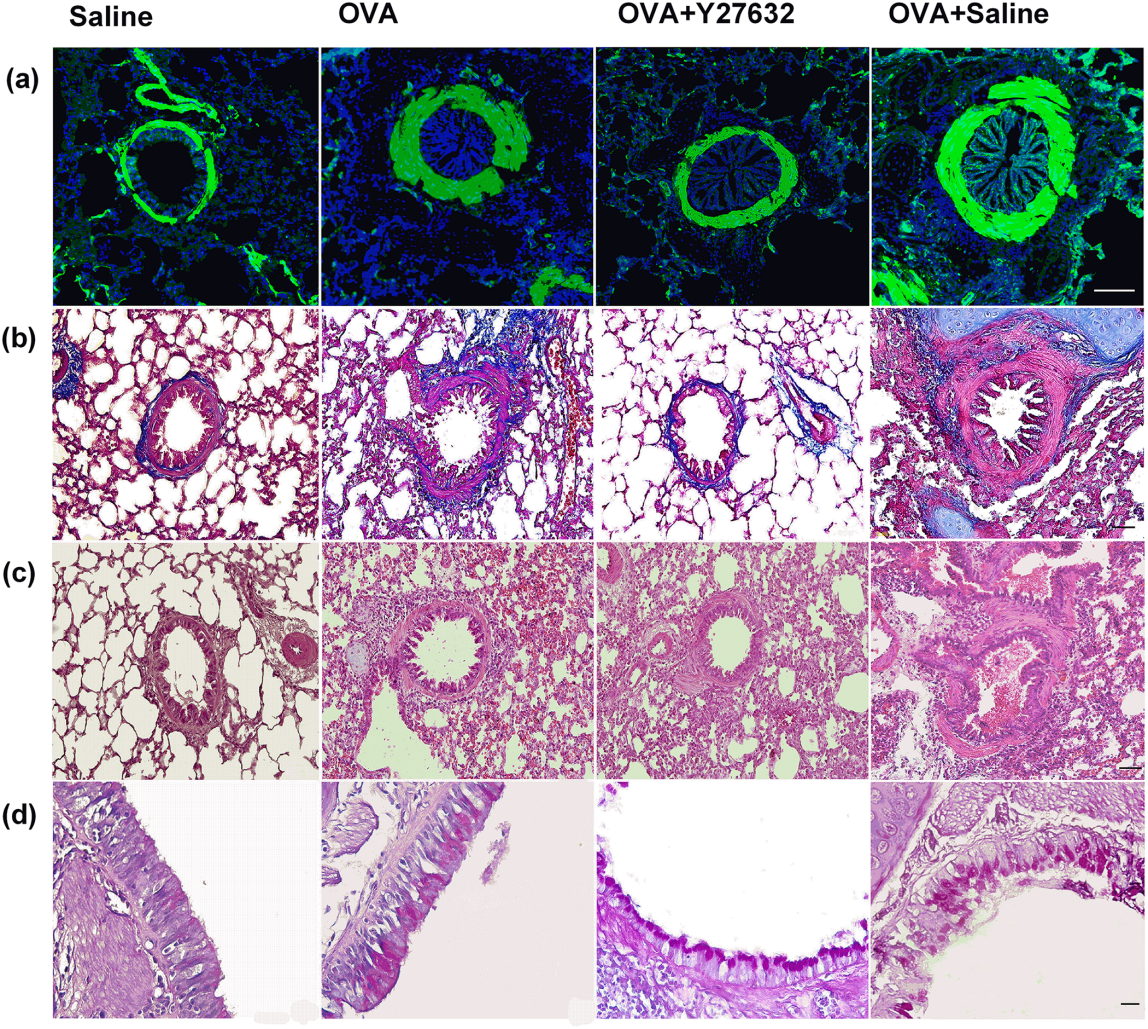
Effect of Rho-kinase inhibition on airway remodeling and inflammation. (a), immunohistofluorescence staining to illustrate expression of α-SMA (Scale bar 100 μm); (b), Masson trichrome staining to identify subepithelial collagen deposition (Scale bar 50 μm); (c), hematoxylin and eosin (H&E) staining to detect peribronchial inflammatory cell infiltration (Scale bar 50 μm). (d), periodic acid-Schiff (PAS) staining to assess epithelial goblet cells (Scale bar 20 μm).

The OVA-challenged guinea pigs displayed significantly higher levels of AHR (p<0.01) compared to saline group (Fig 3b). Inhibition of Rho-kinase in OVA-exposed animals (OVA+Y27632) resulted in reduced AHR (p<0.05) compared to OVA group (Fig 3b). The histological stained sections showed airway goblet cell hyperplasia and a noticeable increase in collagen and α-SMA expression surrounding the airways in OVA group (Fig 4). The mentioned increase was not observed in OVA+Y27632 group, however, goblet cell hyperplasia was similarly occurred in the two groups (Fig 4).

### Effect of Rho-kinase inhibition on lung inflammation

**Fig 5:**
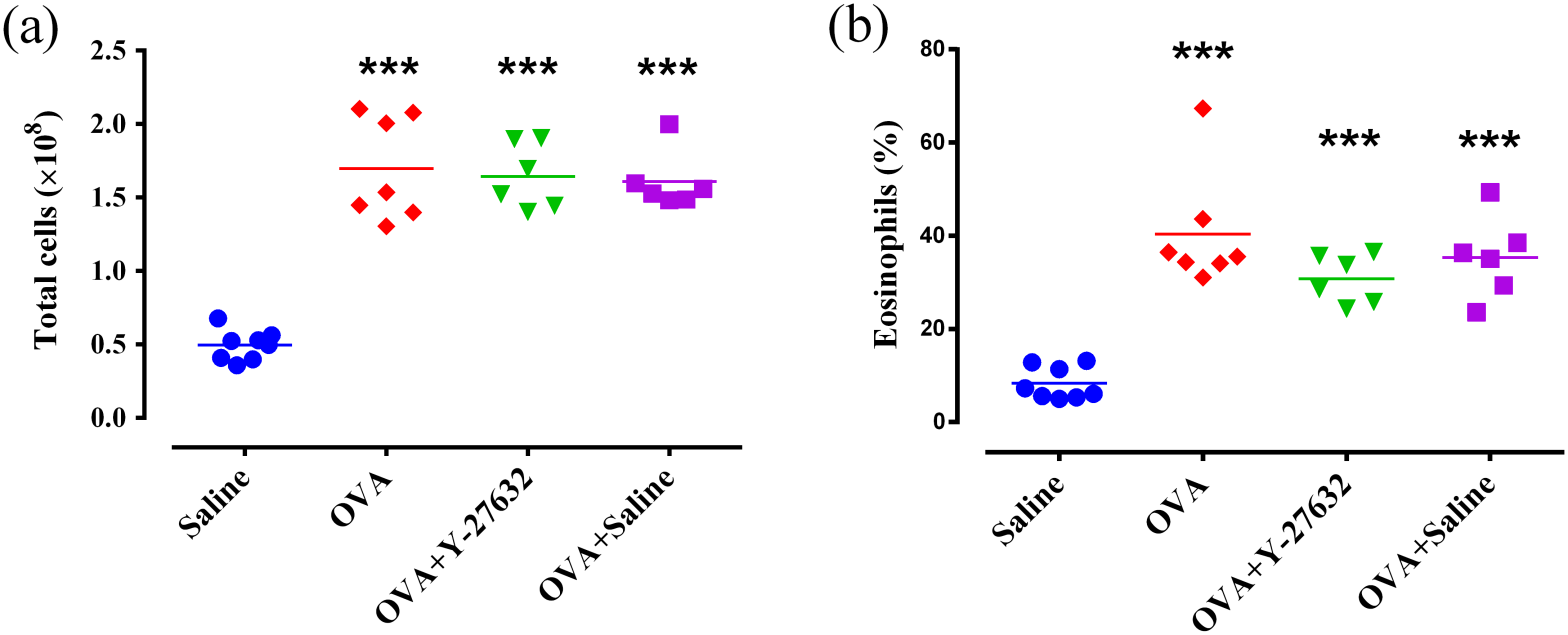
Effect of Rho-kinase inhibition on bronchoalveolar lavage (BAL) inflammatory cells. Total inflammatory cell (a) and eosinophils (b). *** p<0.001 compared to saline. Data were analyzed by a one-way ANOVA, with the Bonferroni post-test.

In OVA group, the levels of total BAL inflammatory cells (p<0.001) and eosinophils (p<0.001) were significantly increased compared to saline group (Fig 5). However, there was no significant difference in total BAL inflammatory cells (p>0.05) and eosinophils (p>0.05) between OVA and OVA+Y27632 groups (Fig 5). Moreover, the H&E staining revealed profound peribronchial inflammatory cell infiltration in OVA and OVA+Y27632 groups (Fig 4).

**Fig 6:**
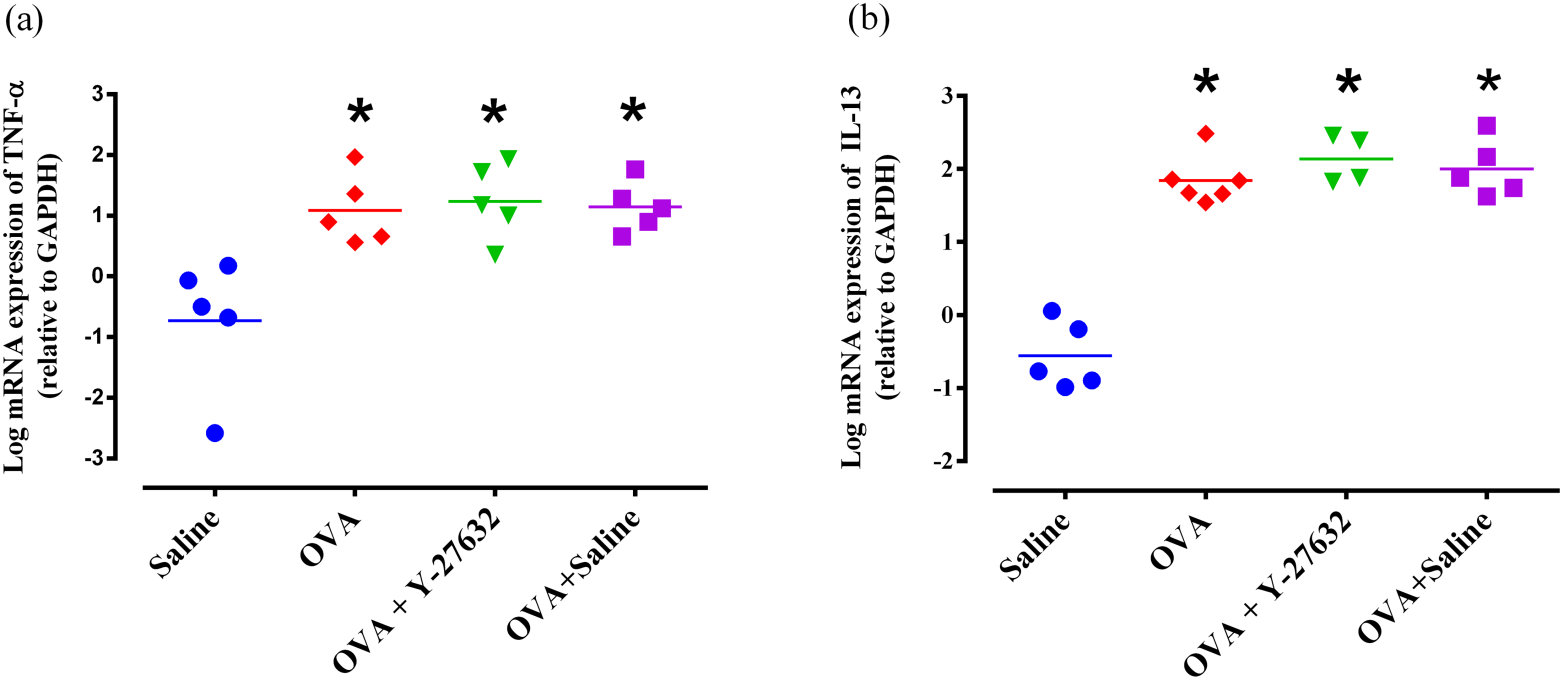
mRNA expression of TNF-α (a) and IL-13 (b) in the lung of experimental groups. * p<0.05 and ** p<0.01 compared to Saline group. Data were analyzed by a Kruskal–Wallis test with a Dunn’s post-test.

Also, repeated exposure to OVA significantly increased the mRNA expression of IL-13 (P<0.05) and TNF-α (P<0.05) in the lung tissue (Fig 6). There was no significant difference in mRNA expression of IL-13 and TNF-α between OVA and OVA+ Y-27632 groups (Fig 6).

### Breathing pattern analysis

**Fig 7:**
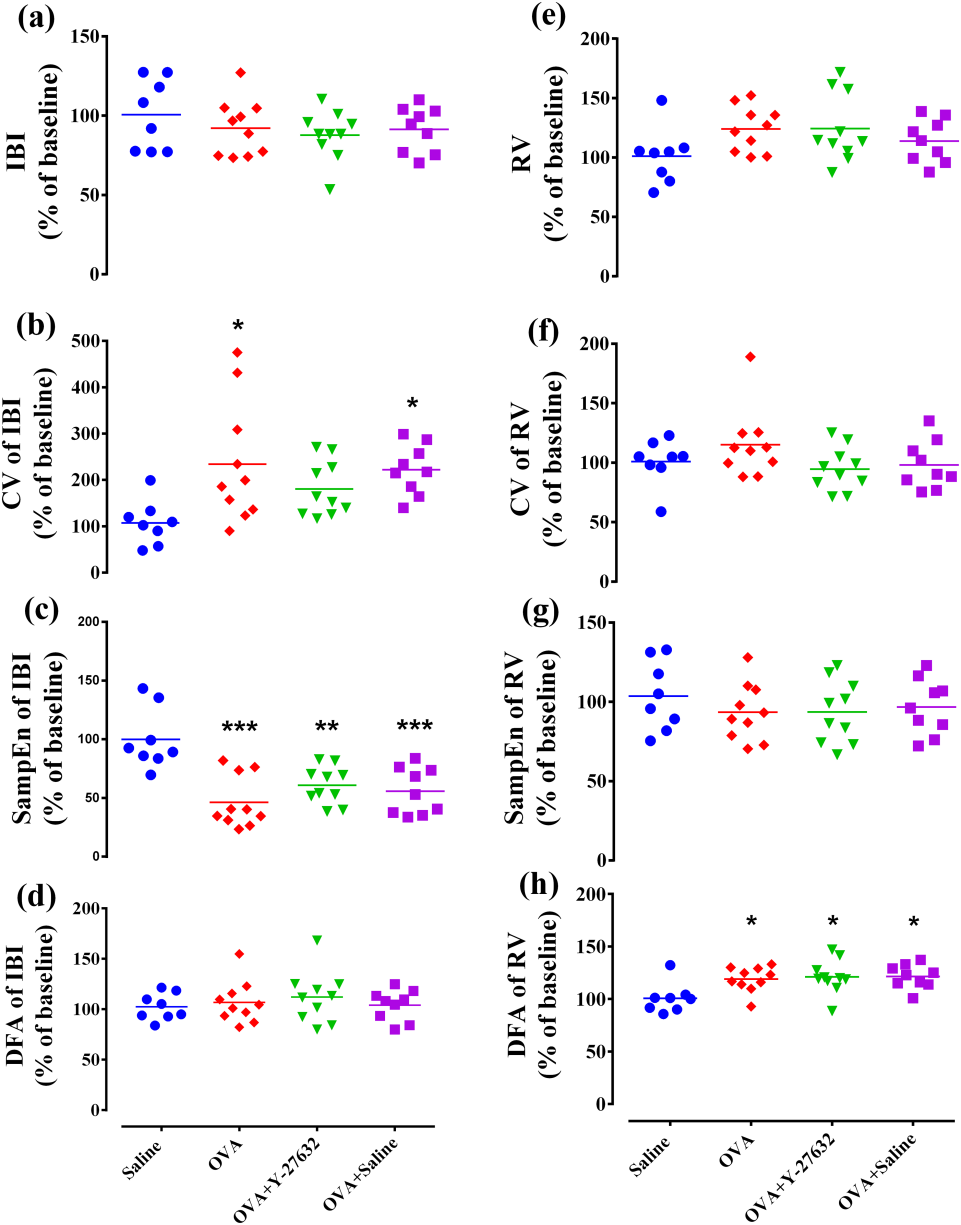
Effect of Rho-kinase inhibition on complexity of respiratory dynamics. * p<0.05, ** p<0.01, and *** p<0.001 compared to saline group. Data were analyzed by one-way ANOVA with the Bonferroni post-test. CV, coefficient of variation; DFA, Detrended fluctuation analysis; SampEn, sample entropy; IBI, inter-breath interval; RV, respiratory volume.

As shown in Fig 5, there were no differences in the mean of IBI and RV among the groups (p > 0.05) (Fig 6a and 6e). However, the CV_IBI_ of OVA and OVA+saline groups were significantly higher than that of saline group (p < 0.05) (Fig 7b). Also, there was no significant difference in the CV_RV_ among the experimental groups (p > 0.05) (Fig 7f). The SampEn_IBI_, as an index of the irregularity, showed a significant decrease in OVA, OVA+saline and OVA+Y27632 groups in comparison with saline (p < 0.001, p<0.001 and p<0.01, respectively) (Fig 7c). However, no significant difference was observed in SampEn_RV_ among the experimental groups (p > 0.05) (Fig 7g). The slope of DFA_RV_ analysis, as an index of self-similarity, in the OVA, OVA+saline and OVA+Y27632 groups was higher than saline group (P < 0.05) (Fig 7h). However, DFA_IBI_ time-series revealed no significant difference among the experimental groups (p > 0.05) (Fig 7d).

**Fig 8:**
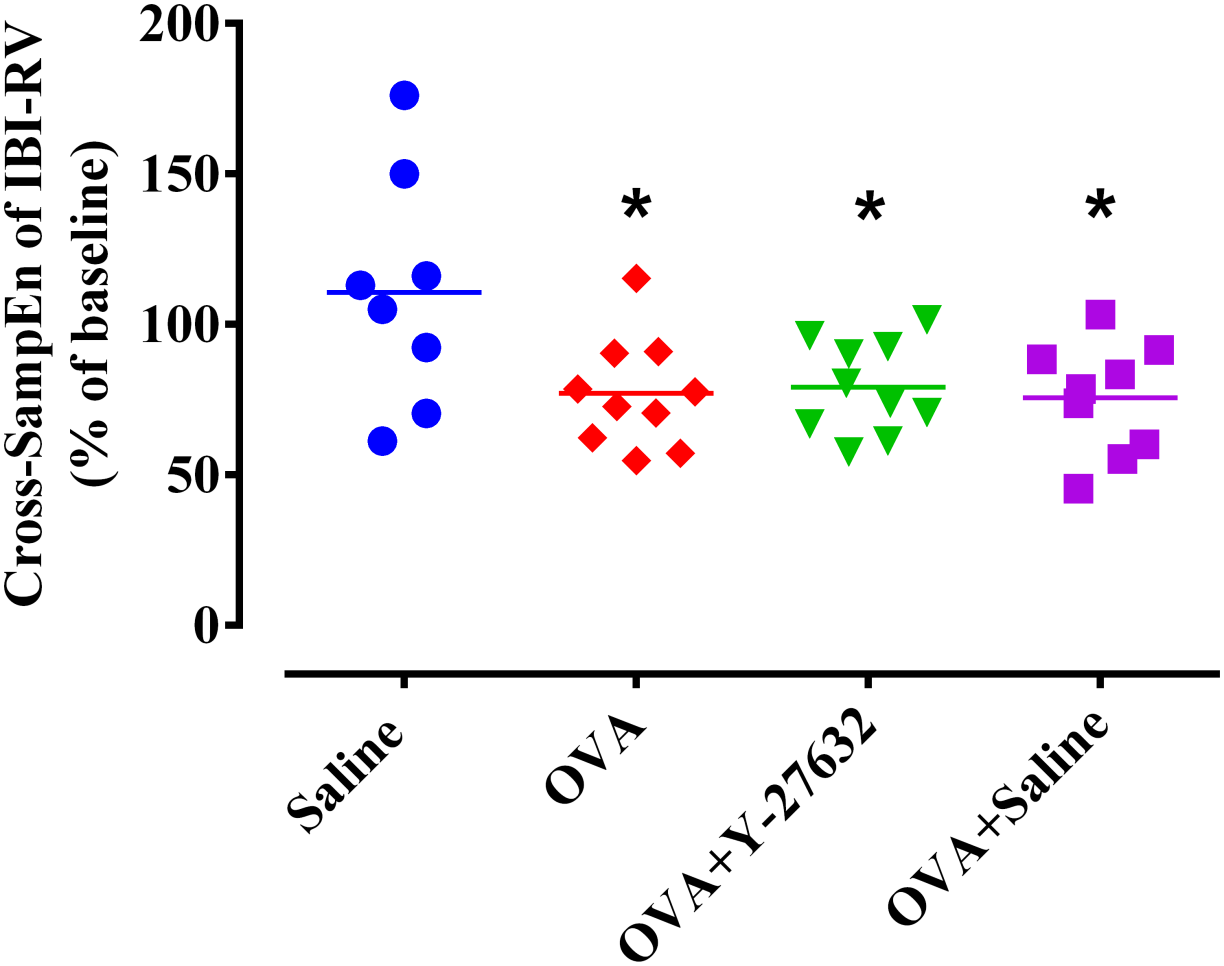
Effect of Rho-kinase inhibition on synchronization between IBI and RV. * p<0.01 compared to saline group. Data was analyzed by one-way ANOVA with the Bonferroni post-test. IBI, inter-breath interval; RV, respiratory volume; Cross-SampEn, cross-sample entropy.

Moreover, we found an increased synchronization between IBI and RV time-series in OVA OVA+saline and OVA+Y-27632 groups using cross-SampEn_IBI-LV_ analysis (p < 0.05) (Fig 8).

## Discussion

In this study, we investigated the effect of Rho-kinase inhibition on nonlinear fluctuation of breathing pattern in a guinea pig model of asthma. Our results showed that the regularity of IBI and long-range correlation (α exponent) of RV increases in asthmatic guinea pigs. Inhibition of Rho-kinase reduced airway remodeling and hyperresponsiveness, but did not affect lung inflammation and complexity of respiratory dynamics in asthmatic animals.

Asthma is considered as a complex disorder with variable clinical behavior [8]. This variability in behavior and clinical symptoms might be associated with greater distance from normal fluctuation and decreased complexity of respiratory dynamics [7, 9]. The complexity analysis of such respiratory fluctuations is non-invasive and enables us to investigate the integrity of respiratory control system. Furthermore, it has the potential to predict controllability, severity and prognosis of asthma [15, 16]. In an experimental setting, analysis of respiratory dynamics requires the respiratory signal be recorded continuously in conscious animals under minimal stressful condition. Moreover, complexity analysis gives insight about the control system rather than detailed molecular mechanism of asthma. However, animal model studies can help to understand the origin of this shift in respiratory dynamics during pathological conditions.

We evaluated respiratory dynamics in asthmatic animal using complexity analysis of both respiratory rate and volume. Reduced SampEn value in IBI time-series of asthmatic guinea pigs indicates higher regularity or lower complexity of the control system. This is consistent with decreased respiratory pattern complexity in patients with asthma [9]. Lower entropy in respiratory dynamics suggests that asthma might be associated with greater distance from normal fluctuation [44]. In addition, greater regularity corresponds to increased isolation of respiratory components from the surrounding environment representing that respiratory system is less adaptive to the external fluctuations [34]. DFA analysis of respiratory signals showed increased long-range correlation in RV time series of asthmatic guinea pigs. This indicates the increased correlation of current fluctuations’ amplitudes with potentially future values. This finding is in contradiction with a previous study that reported decreased long-range correlation RV time series in patients with asthma particularly with uncontrolled asthma state [9]. However, there is a disagreement for optimal value of α exponent. Frey et al. showed increased variability and decreased α exponent of PEF in asthmatic patients associated with increased the risk of asthma exacerbation [11]. On the other hand, enhanced variability and α exponent of PEF has been shown to be associated with failure of treatment in asthmatic patients [12]. Thamrin et al. reported that value of α exponent is related to the level of asthma severity. They suggested that greater α exponent in subjects with mild to moderate asthma is linked to higher possibility of the exacerbation occurrence [45]. Therefore, higher α exponent does not necessarily represent better asthma controllability. Although some previous studies have proposed an optimum value for α exponent [7, 11], there is a report suggesting the possibility of an optimum range (rather than a value) for this factor [12]. This raises the possibility that lower α exponent is associated with too uncorrelated and unpredictable fluctuations in lung function and higher α exponent represents too rigid and deterministic functionality as well [12, 45]. We also found increased respiratory variability of IBI (CV_IBI_) in asthmatic animals. It may be related to unstable respiratory function which may in turn be suggestive of poor disease control [12]. A previous study showed that the increased variability of PEF is associated with augmented unstable lung function [11] and treatment failure in asthmatic patients [12]. However, the precise pathophysiological mechanisms underlying decreased complexity and chaotic nature of respiratory dynamics in asthma is not still thoroughly unveiled.

Asthma is a heterogeneous disease defined by particular clinical and pathological characteristics [46]. Major pathophysiological features of asthma include AHR, airway remodeling, and inflammation [17–19], which are all associated with asthma severity and worse long-term clinical outcomes. Results obtained from animal models indicate that Rho-kinase has a main role in the pathophysiology of asthma [20–23]. A previous study demonstrated that challenge with OVA can induce Rho-kinase activation [23] that is associated with increased collagen synthesis [47], α-SMA expression and fiber assembly, F-actin polymerization [48] and myosin filament assembly in myofibroblasts [49]. We found that the inhibition of Rho-kinase decreases AHR and airway remodeling in asthmatic animal. This finding is in line with the results of previous works that used Rho-kinase inhibitor treatment in asthmatic guiana pigs [28, 29, 50] or mice haploinsufficient for Rho-kinase [27]. Also, in agreement with Kasahara et al. study [27], our results showed that inhibition of Rho-kinase had no significant effect on goblet cell hyperplasia and lung inflammation.

Although Rho-kinase inhibition decreased AHR and airway remodeling that are related to respiratory mechanic, these changes were not effective on complexity of respiratory dynamics. This finding is in parallel to previous studies that provided arguments against the contribution of the mechanical properties of the respiratory system to ventilatory chaos [51, 52]. For instance, in a research by Samara et al., it was observed that breathing against inspiratory threshold and resistive loading in awake humans does not alter the complexity of breathing pattern [52]. Altogether, it seems that changes in respiratory mechanics do not effectively influence the complexity of respiratory dynamics. However, it could be useful to measure the resistance and the compliance of the lungs using an invasive technique in order to validate these results.

It has been postulated that asthma is associated with chronic inflammation of the lung, and some of their symptoms can be due to the influence of inflammation on the nervous system [53]. Experimental evidence has shown that peripheral inflammation associated with chronic airway-induced allergy model alters the immunologic state of the central nervous system (CNS) such as increased intracerebral levels of IgG and IgE [54]. In addition, in a primate model of allergic asthma, it has been shown that prolonged allergen exposure induces neuroplasticity in the hippocampus and nucleus tractus solitarius [53]. These changes in the CNS have been suggested to exaggerate the symptoms in asthmatic patients [53]. Therefore, in addition to changes in respiratory mechanics, it is likely that alterations of central respiratory control system due to airway inflammation may lead to de-complexification of respiratory pattern in asthma. However, we did not examine the effect of airway inflammation on central or peripheral neural activity in the present study, and these issue remains to be investigated in future studies.

Asthma is highly complex, characterized by a fluctuation in inflammatory, structural and functional abnormalities over time in response to different stimuli [8]. Since unidimensional approach restricts the assessment of asthma severity and control, multidimensional approach is suggested as a possible method to address the complexity of the disease [55]. In addition, although traditional reductionist approaches have increased our insight into the mechanism of bronchial constriction/inflammation in asthma, complex systems approaches provide a better understanding of the behavior of the respiratory system as a whole [16]. Such integrative approaches can be used to study the collective behavior of respiratory systems over time and in a multidimensional manner [16]. Complexity analysis of respiratory dynamics considers a set of interacting subunits (eg, inflammatory, neural, and mechanical) in respiratory systems and makes it possible to evaluate the integrity of the whole system [15, 55]. Several studies have suggested that such analysis can provide an additional dimension when compared to traditional assessments of lung function by improving our ability to evaluate asthma severity and control, as well as predict treatment failure [9–12]. Of particular interest is understanding the underlying mechanisms of alteration in breathing pattern complexity in asthma to improve our insights to develop novel interventions and therapeutic strategies [9, 16]. Although our results showed that changes in airway remodeling and hyperresponsiveness do not effectively influence the complexity of respiratory dynamics, further investigations are needed to clarify the main pathophysiological origins of breathing pattern de-complexification in asthma.

## Conclusion

We found reduced the complexity of breathing pattern in guinea pig model of asthma. Inhibition of Rho-kinase improved the airway remodeling and hyperresponsiveness, but had no effect on lung inflammation. Although improved airway remodeling and hyperresponsiveness did not affect the complexity of respiratory dynamics, the effect of respiratory mechanic on breathing pattern complexity should be further investigated using an invasive technique in order to validate these results. Moreover, it seems that inflammation might be involved in alteration of respiratory dynamics in asthma. However, the physiologic meaning of breathing pattern alterations remains exciting avenues of research. Therefore, further studies, with specific focus on role of airway inflammation in respiratory control network, are required to elucidate the mechanisms involved in decreased complexity of breathing pattern in asthma.
